# Influence of genetic polymorphisms in homocysteine and lipid metabolism systems on antidepressant drug response

**DOI:** 10.1186/s12888-020-02798-4

**Published:** 2020-08-14

**Authors:** Baoyu Yuan, Xiaoyan Sun, Zhi Xu, Mengjia Pu, Yonggui Yuan, Zhijun Zhang

**Affiliations:** 1 Department of Neurology, Affiliated ZhongDa Hospital, School of Medical, Southeast University, No.87 Ding Jia Qiao Road, Nanjing, 210009 Jiangsu China; 2 Institution of Neuropsychiatry, Southeast University, Nanjing, 210009 Jiangsu China; 3 Department of Psychosomatics and Psychiatry, Affiliated ZhongDa Hospital Zhongda Hospital, School of Medicine, Southeast University, Nanjing, 210009 Jiangsu China

**Keywords:** Depression, Antidepressant, *MTHFR*, *ApoE*, *ApoA4*, Genetic polymorphism

## Abstract

**Background:**

Variation in genes implicated in homocysteine and lipid metabolism systems may influence antidepressant response for patients with major depressive disorder (MDD). This study aimed to investigate whether association of polymorphisms on the *MTHFR*, *ApoE* and *ApoA4* genes with the treatment response in MDD subjects.

**Methods:**

A total of 281 Han Chinese MDD patients received a single antidepressant drug (SSRI or SNRI) for at least 6 weeks, among whom 275 were followed up for 8 weeks. Their response to 6 weeks’ treatment and remission to 8 weeks’ treatment with antidepressant drugs was determined by changes in the 17-item Hamilton Depression Rating Scale (HARS-17) score. Single SNP and haplotype associations with treatment response were analyzed by UNPHASED 3.0.13. Logistic regression analysis was used to explore the interactions between genotypes and gender or drug type on treatment outcome, only those SNPs that had interactional association with gender or drug type were subjected to further stratified analysis.

**Results:**

In total group, the haplotype (C-A) in *MTHFR* (rsl801133 and rs1801131) and the *ApoE* rs405509 AA genotype were significantly associated with better efficacy of antidepressants; In gender subgroups, only haplotype (C-A) in *MTHFR* (rsl801133 and rs1801131) was significantly associated with better efficacy of antidepressants in male subgroup; In drug type subgroup, the haplotype (C-A) in *MTHFR* (rsl801133 and rs1801131) and haplotype (G-C) in *ApoE* (rs7412 and rs405509) were associated with better efficacy of antidepressants in SNRI treated subgroup; The *ApoA4* rs5092 G allele and GG genotype were associated with worse efficacy of antidepressants in SNRI treated subgroup.

**Conclusions:**

Genetic polymorphisms in homocysteine and lipid metabolism systems are associated with antidepressant response, particularly for the interactions of the certain genetic with gender or drug type.

## Background

Major depressive disorder (MDD) is a common mental disorder with high rates of morbidity, recurrence, and suicide [1, 2]. Although newer antidepressant drugs are generally well tolerated and relatively effective, only 30–40% of patients achieve full remission [3]. Partial remission results in greater suffering among patients, as well as higher costs [4]. The variability in antidepressant drug response can be attributed to several factors, including genetic and environmental influences [5]. Therefore, several authors have attempted to identify variables that could predict antidepressant response, and have suggested several predictors, including clinical, psychosocial, psychophysiological, neuropsychological, neuroimaging, and genetic markers. It has also been suggested that combinations of these variables may improve predictions of treatment response [6–8]. Meta-analyses and consensus suggested that genetic factors are thought to play a pivotal role in individual responses to antidepressant treatment [9, 10]. Genetic research generally focuses on polymorphisms of target proteins, which are related to the mechanisms of action of antidepressant drugs.

Several studies have shown high prevalence rates of folate deficiency in depression [11, 12], presumably because of its impact on neurotransmitter synthesis, which relies on the folate-dependent one-carbon pathway. Low folate level may dampen antidepressant response, increase the risk of depressive relapse, and delay improvement in individuals treated with antidepressants [13]. Folate supplementation appears to improve the response to selective serotonin reuptake inhibitors (SSRIs) [14, 15]. One particular focus with respect to the connection between folate and depression has been the enzyme methylenetetrahydrofolate reductase (MTHFR) [16], which synthesizes 5-methyltetrahydrofolate, a carbon donor involved in the methylation of homocysteine (Hcy) to methionine. This enzyme is encoded by the *MTHFR* gene on chromosome 1 locus *q36.3* in humans. A1298C missense mutation (cytosine-to-thymine) in the *MTHFR* gene results in an alanine-to valine substitution that renders MTHFR thermolabile, and may lead to elevated plasma Hcy, a vascular risk factor [17]. Many recent studies on vascular depression have suggested that chronic lesions in small blood vessels and capillaries could play a role in the pathogenesis of depression [18]. Furthermore, various lines of research have suggested *MTHFR* polymorphisms might enhance the environmental risks (such as low folate intake) for MDD via the interaction between genetic and environmental factors [19]. Several studies performed the association between *MTHFR* polymorphisms and antidepressant treatment response in different populations, which the results were contradictory [20–24]. Therefore, the first purpose of this study was to investigate how *MTHFR* polymorphisms affect antidepressant efficacy in Chinese Han MDD population.

Apolipoproteins (Apo) are lipid-binding proteins involved in the transport of lipids in plasma. Several studies suggested that changes in serum lipid composition may be related to MDD [25]. ApoE is essential for the normal catabolism of triglyceride-rich lipoprotein constituents. Accumulating evidence indicates that *ApoE* polymorphism affects multiple physiopathological pathways in coronary heart disease and Alzheimer’s disease (AD) [26, 27]. *ApoE* including epsilon 2 (ε2), ε3, and ε4 alleles, is encoded by a polymorphic gene located on chromosome 19 [28]. Although protective effects of *ApoE* ε2 have been reported in MDD, and *ApoE* ε4 may be associated with late-onset depression [29], the conclusions of previous studies were not in complete agreement. The present study focused on the ApoE gene promoter region and coding region to investigate the relationships between polymorphic loci and antidepressant efficacy. ApoA4 is another protein involved in lipid metabolic regulation, and has been shown to activate lecithin-cholesterol acyltransferase and cholesteryl esters transfer protein [30]. Data-driven analysis showed that ApoA4 has very high accuracy for discriminating individuals with remitted late-life depression (LLD) compared to never-depressed control participants [31].

A single genetic variant cannot explain the consistent variability observed in patient response to psychiatric treatment [32]. Multiple genetic variations in the Hcy and lipid metabolism pathways could explain more of the variance than a single genetic polymorphism. Therefore, in the present study, we enrolled highly homogeneous MDD patients, evaluated the efficacy of antidepressant therapy at 6 weeks response (primary endpoint) and 8 weeks remission (second endpoint) after received a single antidepressant drug, intended to confirm the association of *MTHFR*, *ApoE* and *ApoA4* with MDD and antidepressant response.

## Methods

### Subjects

**Fig. 1:**
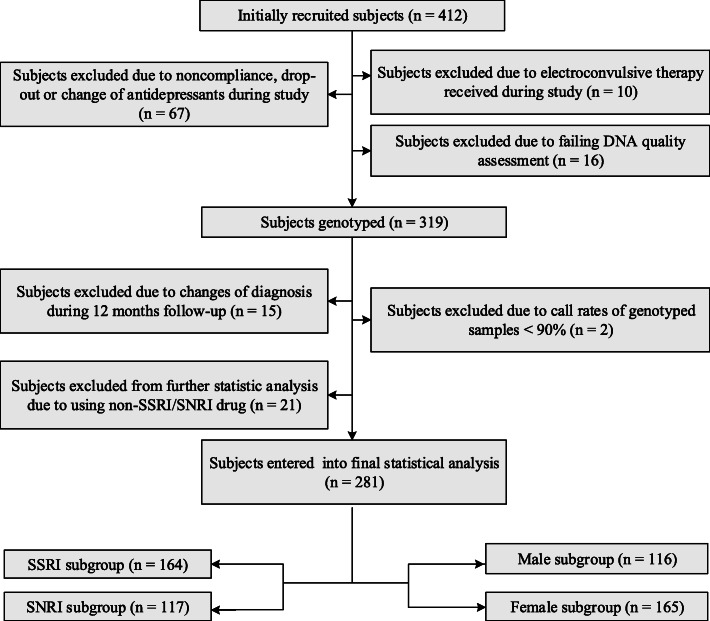
Flow chart of recruited subjects. Abbreviations: SNRI, serotonin norepinephrine reuptake inhibitor; SSRI, selective serotonin reuptake inhibitor

The subjects were Han Chinese inpatients from the database of Institution of Neuropsychiatry, Southeast University. Detailed information of the database referred to our previous study [33]. All patients were fulfilled the criteria for a diagnosis of MDD according to the Diagnostic and Statistical Manual of the American Psychiatric Association (DSM-IV) [34]. The inclusion and exclusion criteria (see details in SI Methods S.1) used to choose subjects can be found in our previously published study [33]. Written informed consent was obtained from all subjects or guardian participants, and the study was approved by the ethics committee of ZhongDa Hospital affiliated to Southeast University or other hospitals participating in the study. A flow chart of recruiting subjects is provided in Fig. 1.

### Antidepressant treatment and clinical evaluation

According to clinical practice guidelines, a total of 281 Han Chinese patients received a single antidepressant drug (SSRI or SNRI) at least 6 weeks, among whom 275 were followed up for 8 weeks. The detail antidepressant treatment and clinical evaluation are provided in SI Methods S.2 and in our previously published study [33].

### Gene selection and genotyping methods

Three candidate genes were selected based on evidence for the involvement of vascular risk factors and lipid metabolism in the mechanism of depression, including *MTHFR*, *ApoE*, and *ApoA4* genes. Eight single nucleotide polymorphisms (SNPs) were detected with minor allele frequency (MAF) values of > 5% in the Asian population, according to the dbSNP and HapMap databases and using gene chips (Table S1). The genotyping methods are provided in SI Methods S.3.

### Statistical analysis

Differences in clinical variables between responder and non-responder groups, as well as remission and non-remission groups, were evaluated by Student’s *t* test or Pearson’s χ^2^ test using SPSS software (version 13.0; SPSS Inc., Chicago, IL). Haploview 4.0 was used to analyze Hardy–Weinberg equilibrium (HWE), MAF, percentage of successful genotyping for each marker (%gene), and linkage disequilibrium (LD; both D’ and *r*^*2*^).

Population power analysis indicates that our sample of 281 subjects has 88.6% power to detect significant (*p* < 0.05) differences between response rates of 71 and 77%, i.e. group differences in the proportion of responders are less than 12.3%. In addition, genetic polymorphisms were associated with therapeutic effects by comparing allele, genotype and haplotype distributions of *MTHFR*、*APOE* and *APOA4* between responders and non-responders, remitters and non-remitters using UNPHASED 3.0.13 (Dudbridge, 2003)). To investigate these relationships in the context of gender and drug type subgroups, we used logistic regression (SPSS 13.0) analysis to explore the interactions between genotypes and gender or drug type on treatment outcome, covariates included age, gender, drug type, and baseline 17-item Hamilton Depression Rating Scale (HDRS-17) score. Only those SNPs that had interactional association with gender or drug type were subjected to further stratified analysis. One thousand random permutations were performed using UNPHASED 3.0.13 software to correct *p*-values for multiple testing in the allelic, genotypic and haplotype association analyses, *P* < 0.05 was considered statistical significance.

## Results

A total of 281 patients completed a 6-week antidepressant treatment course. Among these patients, 205 achieved a response and the response rates about 72.9%. The demographic and clinical characteristics of patients in the responder and non-responder groups are shown in Table 1. There were no significant differences between the 6-week responder and non-responder groups in gender, age, drugs used, years of education, or family history of mood disorders. However, the baseline HDRS-17 score was significantly different between these two groups (*t =* 2.891, *P =* 0.004).

**Table 1:** Demographic characteristics of MDD patients and baseline HDRS-17 scores: comparison between responder and non-responder groups

Demographic characteristics	Responder (*n* = 205)	Non-responder (*n* = 76)	*t /* χ^2^	*P*
Gender (male/female)	81/124	35/41	0.987	0.324
Age (years)	38.99 ± 12.93	36.18 ± 13.36	1.599	0.111
Education (years)	11.27 ± 3.84	12.20 ± 3.82	−1.800	0.073
Family history of mood disorder (yes/no)	29 (14.15%)	15 (19.74%)	−1.031	0.305
Baseline HDRS-17 score	28.18 ± 5.68	26.01 ± 5.32	2.891	**0.004**
Number of episodes	2.01 ± 1.57	2.33 ± 2.03	−1.394	0.164
Antidepressant (SSRI/SNRI)	114/91	50/26	1.569	0.119

*Abbreviations*: *HDRS-17* 17-item Hamilton Depression Rating Scale, *SNRI* serotonin norepinephrine reuptake inhibitor, *SSRI* selective serotonin reuptake inhibitor

A total of 275 patients completed 8-week antidepressant treatment. Among these patients, 144 achieved remission and the remission rates about 52.4%. There were no significant differences in age, number of years of education, family history, baseline HDRS-17 score, or antidepressant agents used between remission and non-remission groups (all *P >* 0.05), while the proportion of male patients and number of episodes were significantly higher in the non-remission group than the remission group (*t =* 2.381, *P =* 0.018 and *t =* − 1.983, *P =* 0.049, respectively), as shown in Tables 1 and 2.

**Table 2:** Demographic characteristics of MDD patients and HDRS-17 scores: comparison between remission and non-remission groups

Demographic characteristics	remission (*n* = 144)	non-remission (*n* = 131)	*t /* χ^2^	*P*
Gender (male/female)	49/95	63/68	2.381	**0.018**
Age (years)	38.38 ± 11.99	38.14 ± 14.11	0.150	0.881
Education (years)	11.28 ± 3.65	11.73 ± 4.07	−0.957	0.340
Family history of mood disorders (yes/no)	20 (13.89%)	22 (16.79%)	−0.645	0.519
Baseline HDRS-17 score	27.03 ± 5.71	28.00 ± 5.36	−1.452	0.148
Number of episodes	1.88 ± 1.30	2.30 ± 2.06	−1.983	**0.049**
Antidepressant (SSRI/SNRI)	76/68	83/48	1.781	0.076

*Abbreviations*: *HDRS-17* 17-item Hamilton Depression Rating Scale, *SNRI* serotonin norepinephrine reuptake inhibitor, *SSRI* selective serotonin reuptake inhibitor

Among the eight SNPs of the three genes investigated, two (*ApoA4* rs5101 and rs675) were eliminated as they had MAF < 5%, while the remaining six SNPs were subjected to further statistical analyses (Table S1). LD analysis showed that two SNPs (rs1801133, rs1801131) of the *MTHFR* gene were in near 100% LD (D’ = 1.0, *r*^*2*^ = 0.177), while two other SNPs (rs405509, rs439401) of the ApoE gene were in strong LD (D’ = 0.961, *r*^*2*^ = 0.505). The other SNPs showed no LD.

An analysis of single locus effects revealed that the *ApoE* rs405509 A allele and AA genotype were significantly associated with the better efficacy of antidepressants at 6 weeks in the total group (A allele: χ2 = 6.27, *P* = 0.012; AA genotype: χ2 = 7.41, *P* = 0.006), but only the AA genotype was significant after permutation testing (P* = 0.04) (Shown in Table 3). The *MTHFR* rs1801133 TT genotype was related to the efficacy of antidepressants, and its distribution frequency was significantly higher in the non-remission group than in the remission group (χ2 = 6.328, *P* = 0.012), but the result did not withstand permutation testing.

**Table 3:** Genetic association analysis (genotypic/allelic) of SNPs versus response status in each subgroups

Groups	SNPs (Gene/rs#)	Allele/Genotype	Response (%)	Non-response (%)	OR (95% CI)	χ^2^	*P*	P*
Total group	ApoE /rs405509	AA	113 (55)	28 (37)	1	7.411	**0.006**	0.04
		AC	82 (40)	42 (55)	0.48 (0.28–0.84)	5.239	**0.022**	
SNRI subgroup	ApoA4/rs5092	A	98 (54)	17 (33)	1	7.241	0.007	0.005
		G	84 (46)	35 (67)	0.42 (0.22–0.80)	7.241	**0.007**	
		AA	23 (25)	2 (8)	1	3.721	0.054	
		AG	62 (54)	32 (64)	0.63 (0.27–1.43)	1.313	0.252	
		GG	16 (18)	11 (42)	0.13 (0.02–0.65)	6.964	**0.008**	0.019
SSRI subgroup	ApoA4 /rs5092	A	124 (54)	52 (52)	1	0.159	0.690	
		G	104 (46)	48 (48)	0.91 (0.57–1.46)	0.159	0.690	
		AA	31 (27)	10 (20)	1	0.959	0.327	
		AG	62 (54)	32 (64)	0.63 (0.27–1.43)	1.313	0.252	
		GG	21 (18)	8 (16)	0.85 (0.29–2.50)	0.14	0.708	

*Abbreviations*: *SNPs* single nucleotide polymorphisms, *SNRI* serotonin norepinephrine reuptake inhibitor, *OR* odds ratio, *CI* confidence interval

*Adjusted *P*-value from 1000 permutation tests

An analysis of haplotype effects demonstrated that the haplotype (C-A) in *MTHFR* (rs1801133 and rs1801131) was significantly associated with antidepressant response in the 8-week antidepressant in the total group (χ2 = 11.39, *P* = 0.0007), the result withstood permutation testing (P* = 0.02) (Shown in Table 4). The haplotypes G-A and G-C (rs7412 and rs405509), C-G (rs405509 and rs439401), and G-C-G (rs7412, rs405509, and rs439401) in *ApoE* were significantly associated with antidepressant response in the 6-week response (rs7412-rs405509: G-A, χ2 = 5.046, *P* = 0.025; G-C, χ2 = 4.313, *P* = 0.038; rs405509-rs439401: χ2 = 5.13, *P* = 0.024; rs7412-rs405509-rs439401: χ2 = 3.907, *P* = 0.048), but the above results did not withstand permutation testing. The non-significant results were included in supplementary materials (Table S2, S3, S4, S5).

**Table 4:** Estimated haplotype frequency of the two MTHFR SNPs (rs1801133 and rs1801131) and results of haplotype analysis in remission and non-remission groups

Group	haplotype	remission (%)	non- remission (%)	*OR (95%CI)*	*χ2*	*P*	P*
Total group	T-A	117 (41)	125 (48)	1	2.795	0.095	
	C-A	127 (44)	79 (30)	1.72 (1.18–2.51)	11.39	**0.0007**	**0.002**
	C-C	44 (15)	58 (22)	0.81 (0.51–1.29)	4.273	0.039	
Male subgroup	T-A	40 (41)	60 (48)	1	1.032	0.310	
	C-A	46 (47)	35 (28)	1.97 (1.09–3.57)	8.767	**0.003**	**0.012**
	C-C	12 (12)	31 (25)	0.58 (0.27–1.26)	5.428	**0.020**	**> 0.05**
Femal subgroup	T-A	77 (41)	65 (48)	1	1.703	0.192	
	C-A	81 (43)	44 (32)	1.55 (0.95–2.55)	3.542	0.060	
	C-C	32 (17)	27 (20)	1 (0.54–1.84)	0.485	0.486	
SSRI subgroup	T-A	59 (39)	74 (45)	1	1.083	0.298	
	C-A	63 (41)	58 (33)	1.46 (0.89–2.41)	2.713	0.100	
	C-C	30 (20)	38 (23)	0.99 (0.55–1.78)	0.470	0.493	
SNRI subgroup	T-A	58 (43)	51 (53)	1	2.48	0.115	
	C-A	64 (47)	25 (26)	2.25 (1.24–4.09)	10.51	**0.001**	**0.002**
	C-C	14 (10)	20 (21)	0.62 (0.28–1.34)	4.998	**0.025**	**> 0.05**

*Abbreviations*: *SNRI* serotonin noradrenaline reuptake inhibitor, *OR* odds ratio, *CI* confidence interval

*Adjusted P-value from 1000 permutation tests

In the logistic regression analyses of the interaction of genotype or haplotype with gender, only the haplotype of *MTHFR* SNPs (rs1801133 and rs1801131) showed significant result (*P* = 0.018, OR = 2.296), the result was shown in supplementary materials (Table S7). This haplotypes of *MTHFR* SNPs (rs1801133 and rs1801131) was further explored within the male/female subgroups; only the haplotype C-A was associated with treatment response in the male subgroup (χ2 = 8.767, *P* = 0.003), the result withstood permutation testing (P* = 0.012) (Table 4).

An exploration of the genotype or haplotype and antidepressant drug type interaction revealed that the SNP of *ApoA4* rs5092, the haplotype of *MTHFR* SNPs (rs1801133 and rs1801131) and the haplotype of *ApoE* SNPs (rs7412 and rs405509) showed significant result (*ApoA4* rs5092: *P* = 0.022, OR = 0.437; haplotype of *MTHFR*: *P* = 0.005; OR = 0.374; haplotype of *ApoE*: *P* = 0.013, OR = 0.399). The above results were shown in supplementary materials (Table S6, S7, S8). Then, the three markers were further investigated in SSRI/SNRI subgroups. As shown in Table 3, the *ApoA4* rs5092 G allele and GG genotype were associated with antidepressant response, of which the antidepressant effect of G allele carriers and GG genotype were poor in SNRI subgroup (G allele: χ2 = 7.241, *P* = 0.007; GG genotype: χ2 = 6.964, *P* = 0.008), the results withstood permutation testing (G allele: P* = 0.005, GG genotype: P* = 0.019). The haplotype C-A was associated with treatment response in the SNRI subgroup (χ2 = 10.51, *P* = 0.001), the result withstood permutation testing (P* = 0.002) (Table 4). As shown in Table 5, the haplotype G-C in *ApoE* (rs7412 and rs405509) was significantly associated with antidepressant response in the SNRI subgroup (χ2 = 8.24, *P* = 0.0041). In comparison with the A-A haplotype, the G-C haplotype was associated with increased likelihood of a better response (OR = 1.04, 95% CI = 0.19–5.64), the result withstood permutations (P* = 0.049).

**Table 5:** Estimated haplotype frequencies of the two ApoE SNPs (rs7412 and rs405509) and the results of haplotype analysis in responders and non-responders

Group	haplotype	response (%)	non-response (%)	*OR (95%CI)*	*χ2*	*P*	P*
SSRI subgroups	rs7412-rs405509						
	A-C	23 (9)	6 (7)	0.65 (0.05–7.63)	0.605	0.437	
	G-A	169 (68)	54 (65)	0.48 (0.06–4.07)	0.263	0.608	
	G-C	46 (18)	21 (26)	0.33 (0.04–2.66)	2.389	0.122	
SNRI subgroup	rs7412-rs405509						
	A-C	12 (7)	3 (5)	3.12 (0.23–42.27)	0.019	0.890	
	G-A	139 (77)	29 (55)	3.38 (0.57–19.42)	7.658	**0.006**	
	G-C	26 (14)	17 (33)	1.04 (0.19–5.64)	8.24	**0.0041**	0.049

*Abbreviations*: *RM* remission; *NR* non-remission, *SNRI* serotonin noradrenaline reuptake inhibitor, *OR* odds ratio, *CI* confidence interval

*Adjusted P-value from 1000 permutation tests

## Discussion

We investigated the association of genetic variation in folate and lipid metabolism-related genes with antidepressant response in patients with MDD, and found significant effects of single polymorphisms in *MTHFR*, *ApoE,* and *ApoA4.*

In the *MTHFR* gene, C677T (rs1801133) and A1298C (rs1801131) are the most investigated SNPs associated with MDD. The C677T variant results from a single nucleotide substitution at this position, in which cytosine (C) is replaced by thymine (T) resulting a conversion of alanine to valine residue which diminishes the enzyme activity diminishes the enzyme activity. Another common polymorphism is A1298C, in which adenine (A) is replaced by cytosine (C) resulting a conversion of glutamate to alanine, which also diminishes the enzyme activity. In the present study, the haplotypes C-A of rs1801133 and rs1801131 were associated with better antidepressant effects in the whole group, and male or SNRI treated subgroups. This may have been because there are mutations in both T-A and C-C haplotypes that result in decreased MTHFR enzyme activity, increased blood Hcy levels, and decreased folate levels [35]. Bottilieri et al. reported that folic acid supplementation can protect brain function by reducing Hcy [36, 37], and also that increased levels of Hcy and/or decreased levels of folate resulted in decreased levels of S-adenosyl methionine (SAM) in cerebrospinal fluid; meanwhile, SAM as a methyl donor for serotonin (5-HT) and catecholamine pathways exerted significant antidepressant effects and was shown to have better efficacy than imipramine [38]. However, it has been suggested [39] that folic acid and vitamin B12 do not clearly enhance the efficacy of antidepressants, and that the use of folic acid and vitamin B12 can only prevent further increases in Hcy but cannot reduce its level; this may be related to differences among studies in the folic acid and vitamin B12 doses used .

This study further showed that only C-A haplotype carriers in the male but not female subgroup experienced higher antidepressant efficacy. This may have been due to the higher folate concentrations and lower Hcy levels in women [40], which compensates for reduced MTHFR enzyme activity, thus making it difficult to detect the relationship between this gene polymorphism and antidepressant efficacy. In addition, women have higher estrogen levels, and estrogen can also weaken the correlation between *MTHFR* polymorphism and antidepressant efficacy to some extent by lowering Hcy levels [41].

In addition, the efficacy of antidepressants was greater in C-A haplotype carriers in the SNRI subgroup. As we all known, SSRIs mainly selectively inhibit the reuptake of 5-HT by the presynaptic membrane. In contrast to SSRIs, SNRIs have a 5-HT reuptake inhibitory effect, as well as noradrenaline (NE) and mild dopamine (DA) reuptake inhibition. It has been reported that depressed patients with high plasma hcy concentrations have significantly lower concentrations of the CSF monoamine metabolites 5-hydroxyindoleacetic acid (5-HIAA), homovanillic acid (HVA), and 4-hydroxy-3-methoxyphenylethylene (MHPG) suggesting an impairment in the metabolism of 5-HT, DA and NE [42]. Therefore, SNRIs maybe have greater effects against the neurotoxicity associated with a high Hcy level. SSRIs have a single drug target, so there was little difference in efficacy between haplotypes in the SSRI subgroup.

In our present study, we also notice that single locus association analysis has found no association between SNPs rsl801131 and rsl801133 with antidepressant response, in agreement with the findings from several studies. Mei F et al. [23] found that the mutation of *MTHFR* gene C677T polymorphism was significantly associated with the increased risk of PSD, but not with antidepressant treatment response. Mischoulon, D et al. [24] reported that *MTHFR* C677T and *MS* A2756G polymorphism did not affect the antidepressant response of fluoxetine treatment. We speculate that a single SNP has less effect on gene function than a haplotype, which is not enough to affect the efficacy of antidepressants.

The *ApoE* gene plays an important role in regulating lipid metabolism and maintaining cholesterol balance. The SNP, rs7412 (C526T), is a functional site in the *ApoE* gene; it is mutated, resulting in replacement of arginine with cysteine. Which indicated that this polymorphism is closely related to lipid metabolism. Although rs405509 (219A/C), which is located in the promoter region upstream of the gene, has not been confirmed to be a functional site, which showed that its polymorphism is associated with antidepressant efficacy. Therefore, further studies regarding this site are required.

Studies showed that serum cholesterol levels in patients with depression were significantly lower than those in healthy people [43]. Furthermore, Sonawalla et al. reported that serum cholesterol levels have also been shown to be associated with the efficacy of antidepressants [44]. Their study found that patients with depression treated with a standard dose of fluoxetine (20 mg/d) had high serum cholesterol levels (≥ 200 mg/dl); with lower cholesterol levels, the curative effect is diminished, the tendency toward chronic disease is greater, and the possibility of recurrence is higher; related research on refractory depression reached a similar conclusion. The above studies suggested that the effects of the *ApoE* gene polymorphism on the efficacy of antidepressants may be related to the concentration of cholesterol in the body. Excessive cholesterol concentration may affect 5-HT transporters and/or various 5-HT receptors [45]. The function of the 5-HT neurons cytomembrane structure has an adverse effect. As another possible explanation, patients with hypercholesterolemia are more likely to have vascular and anxiety disorders, which would affect the efficacy of antidepressants [46]. However, studies have yielded inconsistent results, suggesting that the incidence of depression is lower in the higher blood lipid state, and that high blood lipids may have certain antidepressant effects. For example, Mase et al. [25] reported that low serum high-density lipoprotein (HDL-C) is a marker of suicidal behavior in depression, and may induce immune or inflammatory reactions in depression. Mischoulon et al. [47] also reported that foods rich in docosahexaenoic acid (DHA) are associated with a lower incidence of depression, while DHA deficiency (e.g., alcoholism and postpartum) is associated with a higher incidence of depression. The discrepancies between the above studies may be related to differences in the subjects and indicators of blood lipid levels.

In addition, studies on the association between the *ApoE* gene and depression and antidepressant efficacy have focused on the common alleles of this gene, ε2, ε3, and ε4, and some studies confirmed that the *ApoE* gene ε4 allele was associated with greater efficacy of antidepressants [48–50]. Bizzarro et al. [51] reported a significant association between AD and rs405509 CC genotypes when exploring the association between AD and an *ApoE* gene promoter, suggesting that rs405509 may play a role in the pathogenesis of AD. Lescai et al. [52] also reported that haplotype A-ε4, consisting of rs4905509 and ε4, can increase the risk of late-onset AD by reducing *ApoE* expression levels. Therefore, we hypothesized that the rs405509 polymorphism may have strong LD with other functional SNPs, such as the ε4 allele, and thus alter the biological function of the *ApoE* gene to influence the efficacy of antidepressants.

The function of rs5092 (29A/G) located in the promoter region of the ApoA4 gene, is still unclear and there have been few studies related to this site. However, in an exploration of gene function, the polymorphism of the *ApoA1/C3/A4* gene cluster on chromosome *11q23–24* was shown to be related to blood lipids. These three genes show a high degree of identity and evolved from the same ancestral gene. The *ApoA1* and *ApoA4* genes have the same transcriptional direction, while *ApoC3* is transcribed in the opposite direction [52]; moreover, its polymorphic variation can lead to hypertriglyceridemia [53]. This site may affect the synthesis of *ApoA4*, and the blood lipid level, by altering transcription of the *ApoA4* gene.

Our study has limitations as follows. Firstly, there was no placebo control group in our present study, it is difficult to exclude such effects and other non-pharmaceutical factors. In further research, we can consider establishing a placebo control group to better observe the efficacy of antidepressants. Second, only three genes polymorphism in Hcy and lipid metabolic pathways were included in the present study, their treatment response may be not enough. Regarding the sites shown to be associated with the efficacy of antidepressants studies with larger sample sizes are needed to verify their accuracy and lay a foundation for personalized medicine. Finally, the reports of efficiency to treatment response due to having one of the genotypes may be affected by prior exposure to treatments for depression. Further research could enroll first episode and drug-naive depression patients to further verify the relationship between genetic polymorphisms and antidepressant drug treatment response.

## Conclusions

This study demonstrates that genetic polymorphisms in homocysteine and lipid metabolism systems are associated with antidepressant response in MDD patients, particularly, with the interactions of the certain genetic with gender or drug type. The results of this study and further pharmacogenomics research may lead to individualized, more reasonable and successful new antidepressant treatment strategies.

## Supplementary information

**Additional file 1.**

## Data Availability

The raw/processed data required to reproduce these findings cannot be shared at this time as the data also forms part of an ongoing study. The de-identified dataset used and/or analysed during the current study are available from the corresponding author on reasonable request.

## Abbreviations

5-HIAA: 5-hydroxyindoleacetic acid
5-HT: 5-hydroxytryptamine, serotonin
AD: Alzheimer’s disease
ApoA4: Apolipoprotein A4
ApoE: Apolipoprotein E
CGI: Clinical Global Impression
DA: Dopamine
DHA: Docosahexaenoic acid
DSM-IV: Diagnostic and Statistical Manual of the American Psychiatric Association
Hcy: Homocysteine
HDL-C: High-density lipoprotein
HDRS: Hamilton Depression Rating Scale
HVA: Homovanillic acid
HWE: Hardy–Weinberg equilibrium
LLD: Late-life depression
MAF: Minor allele frequency
MDD: Major depressive disorder
MHPG: 4-hydroxy-3-methoxyphenylglycol
MTHFR: Methylenetetrahydrofolate reductas
NE: Norepinephrine
SAM: S-adenosyl methionine
SNPs: Single nucleotide polymorphisms
SNRI: Serotonin norepinephrine reuptake inhibitor
SSRIs: Serotonin reuptake inhibitors
TESS: Treatment Emergent Symptom Scale
