# Supplementary material for: Influence of genetic polymorphisms in homocysteine and lipid metabolism systems on antidepressant drug response
Source: BMC Psychiatry. 2020 Aug 14;20:408. doi: 10.1186/s12888-020-02798-4 (PMC7427977; doi:10.1186/s12888-020-02798-4)
Supplement: Supplementary file 1 — Additional file 1. [file 12888_2020_2798_MOESM1_ESM.docx]

Influence of genetic polymorphisms in homocysteine and lipid metabolism systems on antidepressant drug response

**Supplementary Information**

**SI methods**

### S.1 Subjects

All subjects had a new diagnosis, or had recently relapsed, and all were drug-free for over 2 weeks and had a baseline score ≥ 18 on the 17-item Hamilton Depression Rating Scale (HDRS-17) [[1](#_ENREF_1)], having presented with depressive symptoms for at least 2 weeks before entry into the study. The diagnoses were made by two independent senior psychiatrists and were confirmed by a third psychiatrist blinded to the previous evaluations. Exclusion criteria included documented history of a diagnosis on Axis 1 (including substance misuse, schizophrenia, schizoaffective disorder, bipolar disorder, generalized anxiety disorder, panic disorder or obsessive–compulsive disorder) of DSM-IV, personality disorder, mental retardation, pregnancy, lactation, primary organic disease or other illness impairing psychiatric evaluation, or a history of electroconvulsive therapy within the previous 6 months. Newly diagnosed patients were also excluded if they had a manic episode in the 12 months following entry.

### S.2 Antidepressant treatment and clinical evaluation

We interviewed each patient biweekly and recorded treatment duration, dosage, outcome, and compliance, using the HDRS-17 to assess the severity of symptoms and therapeutic efficacy while blinded to patient genotypes. Dose increases of the antidepressant drugs prescribed at baseline were allowed if the patient had not achieved Clinical Global Impression (CGI) change scores indicating “much improved” or “very much improved.” Concomitant psychotropic medications were not permitted, except for low-dose benzodiazepine anxiolytic (alprazolam, 0.4–0.8 mg/day; estazolam, 1–2 mg/day) for alleviation of insomnia. Drug side effects were assessed with the Treatment Emergent Symptom Scale (TESS) (Guy, 1976) every 2 weeks, and drug compliance was also monitored routinely via interviews with nursing staff. “Response” was defined as a reduction of at least 50% in the HDRS-17 total score after 6 weeks of treatment, while “remission” was defined as a total HDRS-17 score ≤ 7 points after 8 weeks of treatment, according to the Guidelines for Biological Treatment of Unipolar Depressive Disorders of the World Federation of Societies of Biological Psychiatry [[2](#_ENREF_2)]. Patients requiring a change in antidepressant drug or demonstrating non-adherence were excluded from the study.

### S.3 genotyping methods

Blood was collected in 5-ml EDTA vacutainers and stored at −80°C until genotyping. Genomic DNA was extracted using an NPK-100 Magextractor-genome kit (Toyobo, Osaka, Japan). After quality assessment, DNA samples (250 ng each) were genotyped by Berkeley Biotech Inc. (Menlo Park, CA) using GoldenGate assays (Illumina Inc., San Diego, CA). All of the SNPs selected for the custom oligo pooled assays had Illumina design scores > 0.6. All of our samples had Illumina 10% GenCall scores > 0.4 and call rates > 90%. Genotype data on SNPs were generated using Beadstudio 3.0 (Illumina) and were exported in Excel format for further analysis.

**Supplementary Tables**

**Table S1. General characteristics of polymorphisms genotyped**

| *gene* | Name | HWpval | %gene | MAF | Alleles |
| --- | --- | --- | --- | --- | --- |
| *MTHFR* | rs1801133 | 0.8488 | 100 | 0.438 | C:T |
| *MTHFR* | rs1801131 | 0.6889 | 100 | 0.185 | A:C |
| *APOE* | rs7412 | 0.4691 | 100 | 0.11 | G:A |
| *APOE* | rs405509 | 0.1284 | 100 | 0.278 | A:C |
| *APOE* | rs439401 | 0.5776 | 100 | 0.413 | A:G |
| *APOA4* | rs5101 | 1.0 | 99.6 | **0.0** | G:G |
| *APOA4* | rs5092 | 0.037 | 100 | 0.482 | A:G |
| *APOA4* | rs675 | 1.0 | 100 | **0.005** | A:T |

HWpval:the Hardy–Weinberg equilibrium p value.

%gene: the percentage non-missing for this marker.

MAF: the minor allele frequency for this marker.

Alleles: the major and minor alleles for this marker

**Table S2 Comparison of the frequency of *MTHFR/ApoE/ApoA4 SNPs* alleles in the 6-week response and non-response groups**

| *gene* | *SNPs* | Alleles/genotype | response（%） | Non-response（%） | *OR（95%CI）* | *χ^2^* | *P* | *P** |
| --- | --- | --- | --- | --- | --- | --- | --- | --- |
| *MTHFR* | rs1801133 | T | 174（42） | 72（47） | 1 | 1.095 | 0.295 |  |
|  |  | C | 236（58） | 80（53） | 1.22（0.84-1.77） | 1.095 | 0.295 |  |
|  | rs1801131 | A | 334（81） | 124(82) | 1 | 0.001 | 0.975 |  |
|  |  | C | 76（19） | 28（18） | 1.01（0.62-1.63） | 0.001 | 0.975 |  |
| *MTHFR* | rs1801133 | TT | 37（18） | 18（24） | 1 | 1.119 | 0.290 |  |
|  |  | TC | 100（49） | 36（47） | 1.35（0.68-2.67） | 0.044 | 0.833 |  |
|  |  | CC | 68（33） | 22（29） | 1.50（0.72-3.15） | 0.454 | 0.500 |  |
|  | rs1801131 | AA | 136（66） | 52（68） | 1 | 0.108 | 0.742 |  |
|  |  | AC | 62（30） | 20（26） | 1.19（0.65-2.15） | 0.414 | 0.52 |  |
| *ApoE* | rs7412 | A | 43（10） | 19（13） | 1 | 0.457 | 0.499 | **>0.05** |
|  |  | G | 367（90） | 133（88） | 1.22（0.69-2.17） | 0.457 | 0.499 |  |
|  | rs405509 | A | 308（75） | 98（64） | 1 | 6.27 | **0.012** |  |
|  |  | C | 102（25） | 54（36） | 0.60（0.40-0.90） | 6.27 | **0.012** |  |
|  | rs439401 | A | 247（60） | 83（55） | 1 | 1.454 | 0.228 |  |
|  |  | G | 163（40） | 69（45） | 0.79（0.55-1.16） | 1.454 | 0.228 |  |
| *ApoE* | rs7412 | AG | 35（17） | 17（22） | 0.51（0.05-4.97） | 1.031 | 0.310 | **0.04** |
|  |  | GG | 166（81） | 58（76） | 0.72（0.08-6.53） | 0.745 | 0.388 |  |
|  | rs405509 | AA | 113（55） | 28（37） | 1 | 7.411 | **0.006** |  |
|  |  | AC | 82（40） | 42（55） | 0.48（0.28-0.84） | 5.239 | **0.022** |  |
|  | rs439401 | AA | 73（36） | 21（28） | 1 | 1.585 | 0.208 |  |
|  |  | AG | 101（49） | 41（54） | 0.71（0.39-1.30） | 0.486 | 0.486 |  |
|  |  | GG | 31（15） | 14（18） | 0.64（0.29-1.41） | 0.449 | 0.503 |  |
| *ApoA4* | rs5092 | A | 222（54） | 69（45） | 1 | 3.402 | 0.065 |  |
|  |  | G | 188（46） | 83（55） | 0.70（0.48-1.02） | 3.402 | 0.065 |  |
| *ApoA4* | rs5092 | AA | 54（26） | 12（16） | 1 | 3.435 | 0.064 |  |
|  |  | AG | 114（56） | 45（59） | 0.56（0.28-1.15） | 0.293 | 0.589 |  |

**Table S3 Comparison of the frequency of *MTHFR/ ApoE/ ApoA4 SNPs* alleles in the 8-week remission and non- remission groups**

| *gene* | SNPs | alleles | | remission（%） | non- remission（%） | OR（95%CI） | χ2 | P | P* |
| --- | --- | --- | --- | --- | --- | --- | --- | --- | --- |
| *MTHFR* | rs1801133 | T | | 117（41） | 125（48） | 1 | 2.795 | 0.095 |  |
|  |  | C | | 171（59） | 137（52） | 1.33（0.95-1.87） | 2.795 | 0.095 |  |
|  | rs1801131 | A | | 244（85） | 204（73） | 1 | 4.273 | **0.039** | **>0.05** |
|  |  | C | | 44（15） | 58（22） | 0.63（0.41-0.98） | 4.273 | **0.039** |  |
| *MTHFR* | rs1801133 | TT | | 20（14） | 34（26） | 1 | 6.328 | **0.012** | **>0.05** |
|  |  | TC | | 77（53） | 57（44） | 2.30（1.20-4.40） | 2.724 | 0.099 |  |
|  |  | CC | | 47（33） | 40（31） | 2.00（1.00-4.00） | 0.141 | 0.708 |  |
|  | rs1801131 | AA | | 104（72） | 80（61） | 1 | 3.854 | **0.049** | **>0.05** |
|  |  | AC | | 36（25） | 44（34） | 0.63（0.37-1.07） | 2.452 | 0.117 |  |
| *ApoE* | rs7412 | A | | 29（10） | 33（13） | 1 | 0.875 | 0.350 |  |
|  |  | G | | 259（90） | 229（87） | 1.29（0.76-2.19） | 0.875 | 0.350 |  |
|  | rs405509 | A | | 215（75） | 181（69） | 1 | 2.11 | 0.146 |  |
|  |  | C | | 73（25） | 81（31） | 0.76（0.52-1.10） | 2.11 | 0.146 |  |
|  | rs439401 | A | | 170（59） | 154（59） | 1 | 0.004 | 0.953 |  |
|  |  | G | | 118（41） | 108（41） | 0.99（0.70-1.39） | 0.004 | 0.953 |  |
| *ApoE* | rs7412 | AG | | 25（17） | 27（21） | 1.39（0.21-9.01） | 0.472 | 0.492 |  |
|  |  | GG | | 117（81） | 101（77） | 1.74（0.28-10.6） | 0.719 | 0.396 |  |
|  | rs405509 | AA | | 79（55） | 58（44） | 1 | 3.075 | 0.080 |  |
|  |  | AC | | 57（40） | 65（50） | 0.64（0.39-1.05） | 2.799 | 0.094 |  |
|  |  | CC | | 8（6） | 8（6） | 0.73（0.26-2.07） | 0.038 | 0.845 |  |
|  | rs439401 | AA | | 52（36） | 41（31） | 1 | 0.710 | 0.399 |  |
|  |  | AG | | 66（46） | 72（55） | 0.72（0.43-1.23） | 2.286 | 0.131 |  |
|  |  | GG | | 26（18） | 18（14） | 1.14（0.55-2.36） | 0.950 | 0.330 |  |
| *ApoA4* | rs5092 | A | 157（55） | | 128（49） | 1 | 1.76 | 0.185 |  |
|  |  | G | 131（45） | | 134（51） | 0.80（0.57-1.12） | 1.76 | 0.185 |  |
| *ApoA4* | rs5092 | AA | 35（24） | | 29（22） | 1 | 0.181 | 0.671 |  |
|  |  | AG | 87（60） | | 70（53） | 1.03（0.57-1.85） | 1.365 | 0.243 |  |
|  |  | GG | 22（15） | | 32（24） | 0.57（0.27-1.19） | 3.639 | 0.056 |  |

*P** Adjusted p-value from 1000 permutation tests.

**Table S4Comparison of the frequency of *MTHFR/ApoE*haplotype in the 6-week response andnon-response**

| *gene* | haplotype | response（%） | Non-response（%） | *OR（95%CI）* | *χ^2^* | *P* | *P** |
| --- | --- | --- | --- | --- | --- | --- | --- |
| *MTHFR* | T-A | 174（42） | 72（47） | 1 | 1.095 | 0.295 |  |
|  | C-A | 160（39） | 52（34） | 1.27（0.84-1.93） | 1.094 | 0.296 |  |
|  | C-C | 76（19） | 28（18） | 1.12（0.67-1.88） | 0.001 | 0.975 |  |
| *ApoE* | rs7412-rs405509 | | | | | |  |
|  | A-C | 30（7） | 16（10） | 0.43（0.07-2.45） | 1.502 | 0.220 | **>0.05**  **>0.05**  **>0.05**  **>0.05** |
|  | G-A | 295（72） | 95（62） | 0.71（0.15-3.37） | 5.046 | **0.025** |  |
|  | G-C | 72（18） | 38（25） | 0.44（0.10-2.01） | 4.313 | **0.038** |  |
|  | rs7412-rs439401 | | | | | |  |
|  | A-G | 30（7） | 15（10） | 0.60（0.09-3.89） | 0.915 | 0.339 |  |
|  | G-A | 234（57） | 79（57） | 0.90（0.18-4.42） | 1.385 | 0.239 |  |
|  | G-G | 133（33） | 54（33） | 0.75（0.16-3.4） | 0.611 | 0.435 |  |
|  | rs405509-rs439401 | | | | | |  |
|  | A-A | 246（60） | 81（53） | 1 | 2.087 | 0.149 |  |
|  | A-G | 62（15） | 17（11） | 1.18（0.65-2.14） | 1.399 | 0.237 |  |
|  | C-G | 101（25） | 52（34） | 0.64（0.42-0.97） | 5.13 | **0.024** |  |
|  | rs7412-rs405509-rs439401 | | | | | |  |
|  | A-C-G | 30（7） | 15（10） | 0.23（0.02-2.42） | 0.937 | 0.333 |  |
|  | G-A-A | 233（57） | 79（52） | 0.34（0.04-3.13） | 1.346 | 0.246 |  |
|  | G-A-G | 62（15） | 16（11） | 0.44（0.05-4.25） | 1.79 | 0.181 |  |
|  | G-C-G | 71（17） | 36（24） | 0.22（0.03-2.00） | 3.907 | **0.048** |  |

P* Adjusted p-value from 1000 permutation tests.

**Table S5 Comparison of the frequency of MTHFR/ *ApoE*haplotype in the 8-week remission and non- remission groups**

| *gene* | haplotype | | remission（%） | non- remission（%） | *OR（95%CI）* | *χ2* | *P* | *P** |
| --- | --- | --- | --- | --- | --- | --- | --- | --- |
| *MTHFR* | T-A | | 117（41） | 125（48） | 1 | 2.795 | 0.095 | **0.002** |
|  | C-A | | 127（44） | 79（30） | 1.72（1.18-2.51） | 11.39 | **0.0007** |  |
|  | C-C | | 44（15） | 58（22） | 0.81（0.51-1.29） | 4.273 | 0.039 |  |
| *ApoE* | rs7412-rs405509 | | | | | | | |
|  | A-C | 19（7） | | 26（10） | 0.49（0.13-1.91） | 1.891 | 0.169 |  |
|  | G-A | 205（71） | | 174（67） | 0.79（0.25-2.51） | 1.657 | 0.198 |  |
|  | G-C | 54（19） | | 55（21） | 0.67（0.21-2.1） | 0.556 | 0.456 |  |
|  | rs7412-rs439401 | | | | | | | |
|  | A-G | 20（7） | | 25（10） | 0.65（0.15-2.85） | 1.138 | 0.286 |  |
|  | G-A | 161（56） | | 146（56） | 0.92（0.27-3.11） | 0.012 | 0.913 |  |
|  | G-G | 98（34） | | 83（32） | 0.99（0.31-3.21） | 0.278 | 0.598 |  |
|  | rs405509-rs439401 | | | | | | | |
|  | A-A | 169（59） | | 152（58） | 1 | 0.028 | 0.868 |  |
|  | A-G | 46（16） | | 29（11） | 1.42（0.85-2.37） | 2.77 | 0.096 |  |
|  | C-G | 72（25） | | 79（30） | 0.82（0.56-1.21） | 1.818 | 0.178 |  |
|  | rs7412-rs405509-rs439401 | | | | | | | |
|  | A-C-G | 19（7） | | 25（10） | 0.41（0.10-1.72） | 1.498 | 0.221 |  |
|  | G-A-G | 46（16） | | 28（11） | 0.89（0.24-3.27） | 3.033 | 0.082 |  |
|  | G-C-G | 53（18） | | 53（20） | 0.54（0.16-1.84） | 0.535 | 0.465 |  |

Adjusted p-value from 1000 permutation tests.

**Table S6Interaction of ApoA4 SNP rs5092* Genderand rs5092*drug type on the antidepressant response adjusting for age and baseline HDRS score**

|  | β | SE | p | OR(95% CI) |
| --- | --- | --- | --- | --- |
| Gender | 0.117 | 0.656 | 0.859 | 1.124 (0.311-4.062) |
| Age | -0.009 | 0.010 | 0.374 | 0.991 (0.972-1.011) |
| Drug type | 1.168 | 0.655 | 0.074 | 3.217 (0.892-11.608) |
| baseline HDRS score | -0.065 | 0.028 | 0.022 | 0.937 (0.886-0.991) |
| rs5092 | 0.437 | 0.291 | 0.133 | 1.548 (0.875-2.737) |
| rs5092 by Gender | 0.133 | 0.364 | 0.714 | 1.142 (0.560-2.332) |
| **rs5092 by Drugtype** | **-.828** | **0.360** | **0.022** | **0.437 (0.216-0.886)** |

**Table S7Interaction of MTHFRSNPs (rs1801133 and rs1801131) haplotypes* Gender and haplotypes *drug type on the antidepressant remission adjusting for age and baseline HDRS score**

|  | β | SE | p | OR (95% CI) |
| --- | --- | --- | --- | --- |
| Gender | -1.109 | 0.793 | 0.162 | 0.330 (0.070-1.561) |
| Age | -0.001 | 0.009 | 0.908 | 0.999 (0.981-1.017) |
| Drug type | 0.040 | 0.025 | 0.111 | 1.041 (0.991-1.093) |
| baseline HDRS score | 2.426 | 0.768 | 0.002 | 11.309 (2.510-50.947) |
| haplotypes | -0.095 | 0.290 | 0.743 | 0.909 (0.515-1.606) |
| haplotypes by Gender | **0.831** | **0.351** | **0.018** | **2.296 (1.155-4.565)** |
| haplotypes by Drugtype | **-0.985** | **0.351** | **0.005** | **0.374 (0.188-0.743)** |

**Table S8Interaction of ApoE SNPs (rs7412 and rs405509) haplotypes* Gender and haplotypes *drug type on the antidepressant response adjusting for age and baseline HDRS score**

|  | β | SE | p | OR (95% CI) |
| --- | --- | --- | --- | --- |
| Gender | 1.052 | 0.823 | 0.201 | 2.863(0.571-14.362) |
| Age | -0.001 | 0.010 | 0.900 | 0.999(0.979-1.019) |
| Drug type | -0.084 | 0.029 | 0.004 | 0.920(0868-0.974) |
| baseline HDRS score | 1.636 | 0.809 | 0.043 | 5.135(1.053-25.050) |
| haplotypes | 0.453 | 0.322 | 0.159 | 1.573(0837-0955) |
| haplotypes by Gender | -0.299 | 0.371 | 0.420 | 0.741(0.358-1.533) |
| haplotypes by Drugtype | **-0.920** | **0.369** | **0.013** | **0.399(0.193-0.822)** |

**Supplementary reference**

1. Takahashi: **Rating scale for depression**. *Journal of Neurology Neurosurgery & Psychiatry* 1998, **23**(1):: 56–62.

2. Bauer M, Whybrow PC, Angst J, Versiani M, Moller HJ: **World Federation of Societies of Biological Psychiatry (WFSBP) Guidelines for Biological Treatment of Unipolar Depressive Disorders, Part 1: Acute and continuation treatment of major depressive disorder**. *The world journal of biological psychiatry : the official journal of the World Federation of Societies of Biological Psychiatry* 2002, **3**(1):5-43.
